# Pre-existing Immunity to Japanese Encephalitis Virus Alters CD4 T Cell Responses to Zika Virus Inactivated Vaccine

**DOI:** 10.3389/fimmu.2021.640190

**Published:** 2021-02-24

**Authors:** Noemia S. Lima, Damee Moon, Samuel Darko, Rafael A. De La Barrera, Leyi Lin, Michael A. Koren, Richard G. Jarman, Kenneth H. Eckels, Stephen J. Thomas, Nelson L. Michael, Kayvon Modjarrad, Daniel C. Douek, Lydie Trautmann

**Affiliations:** ^1^Human Immunology Section, Vaccine Research Center, National Institute of Allergy and Infectious Diseases, National Institutes of Health, Bethesda, MD, United States; ^2^Cellular Immunology Section, US Military HIV Research Program, Walter Reed Army Institute of Research, Silver Spring, MD, United States; ^3^Henry M. Jackson Foundation for the Advancement of Military Medicine, Bethesda, MD, United States; ^4^Pilot Bioproduction Facility, Walter Reed Army Institute of Research, Silver Spring, MD, United States; ^5^Division of AIDS, National Institute of Allergy and Infectious Diseases, National Institutes of Health, Bethesda, MD, United States; ^6^Viral Disease Branch, Walter Reed Army Institute of Research, Silver Spring, MD, United States; ^7^Division of Infectious Diseases, Department of Medicine, State University of New York Upstate, Syracuse, NY, United States; ^8^Center for Infectious Diseases Research, Walter Reed Army Institute of Research, Silver Spring, MD, United States; ^9^Emerging Infectious Diseases Branch, Walter Reed Army Institute of Research, Silver Spring, MD, United States; ^10^Vaccine and Gene Therapy Institute, Oregon Health & Science University, Beaverton, OR, United States

**Keywords:** zika virus, vaccine, flavivirus, CD4 T cell, cross-reactivity, TCR repertoire

## Abstract

The epidemic spread of Zika virus (ZIKV), associated with devastating neurologic syndromes, has driven the development of multiple ZIKV vaccines candidates. An effective vaccine should induce ZIKV-specific T cell responses, which are shown to improve the establishment of humoral immunity and contribute to viral clearance. Here we investigated how previous immunization against Japanese encephalitis virus (JEV) and yellow fever virus (YFV) influences T cell responses elicited by a Zika purified-inactivated virus (ZPIV) vaccine. We demonstrate that three doses of ZPIV vaccine elicited robust CD4 T cell responses to ZIKV structural proteins, while ZIKV-specific CD4 T cells in pre-immunized individuals with JEV vaccine, but not YFV vaccine, were more durable and directed predominantly toward conserved epitopes, which elicited Th1 and Th2 cytokine production. In addition, T cell receptor repertoire analysis revealed preferential expansion of cross-reactive clonotypes between JEV and ZIKV, suggesting that pre-existing immunity against JEV may prime the establishment of stronger CD4 T cell responses to ZPIV vaccination. These CD4 T cell responses correlated with titers of ZIKV-neutralizing antibodies in the JEV pre-vaccinated group, but not in flavivirus-naïve or YFV pre-vaccinated individuals, suggesting a stronger contribution of CD4 T cells in the generation of neutralizing antibodies in the context of JEV-ZIKV cross-reactivity.

## Introduction

Zika virus (ZIKV) historically caused rare and mild disease in sub-Saharan Africa and the Indian Ocean basin. Several small sporadic outbreaks of ZIKV also occurred, most notably in Micronesia and French Polynesia in 2007 and 2013, respectively. 2015 marked the largest and most rapid geographic expansion of ZIKV, primarily in the tropical and sub-tropical zones of the Western Hemisphere (1, 2). ZIKV infection is commonly asymptomatic or accompanied by mild symptoms such as low-grade fever, rash, myalgia, arthralgia, and conjunctivitis. However, the public health emergency of international concern that the ZIKV outbreak precipitated in 2016 revealed a strong causal association with neurologic complications such as Guillain-Barré syndrome and Congenital Zika Syndrome (CZS) (3, 4). Although the number of ZIKV infection cases has subsequently declined, there remains a significant risk of resurgent outbreaks as population-level immunity wanes and new naïve cohorts emerge (5). Therefore, there is still a need for the development of a safe and effective ZIKV vaccine. Moreover, a safe vaccine that could be administered to pregnant women would not only prevent CZS, but would also provide passive immunity to infants for the first months of life, which is important because ZIKV infection during early infancy can also impair early neurological development (6, 7). Although neutralizing antibody titers correlate with vaccine protection in NHP models (8), there is evidence showing that CD4 T cell responses are required to promote protective humoral responses against ZIKV (9, 10), and that CD8 T cells are necessary for viral clearance (11, 12). Thus, a protective vaccine should induce not only ZIKV-specific antibodies, but also efficient T cell responses.

ZIKV is a mosquito-borne flavivirus, mainly transmitted by the *Aedes aegypti* mosquito (13), yet, other routes such as sexual and vertical transmission also constitute a significant risk of person-to-person spread (14, 15). ZIKV co-circulates with other closely related flaviviruses, such as dengue virus (DENV), yellow fever virus (YFV), West Nile virus (WNV), and Japanese encephalitis virus (JEV) (16), rendering the populations vulnerable to multiple flavivirus infections. In addition to overlapping epidemiology, ZIKV exhibits high antigenic similarity to other flaviviruses. The envelope (E) protein sequence bears approximately 55% amino acid identity with DENV, 50% with JEV, and 40% with YFV (17). Since this protein is the main target for neutralizing antibodies (18) and has also been mapped for immunodominant CD4 and CD8 T cell epitopes (19–22), cross-reactivity among similar epitopes may play an important role in establishing protective immune responses. For instance, DENV-specific T cells have been shown to recognize ZIKV epitopes (11, 23), and ZIKV-specific T cells are elicited earlier and at higher magnitudes in DENV pre-exposed than in DENV-naïve individuals (20). However, limited T cell cross-recognition has been detected in individuals vaccinated against YFV (24). Importantly, immunity to DENV or YFV prior to ZIKV infection in rhesus macaques has resulted in more CD4 T cell activation and higher titers of anti-ZIKV IgG (25).

The existence of licensed vaccines against other flaviviruses has set the ground for the development and testing of new flavivirus vaccine candidates. The live-attenuated virus vaccine against YFV is a gold standard of vaccine efficacy and durability, as it confers lifelong protection in more than 90% of vaccinees. It is known to induce long lasting neutralizing antibodies and robust CD8 and CD4 T cell responses, with a balanced Th1/Th2 profile (26). A recently licensed chimeric tetravalent DENV vaccine uses the live-attenuated YFV as a backbone to express the virion surface proteins, prM and E, from all 4 serotypes of DENV (27). This vaccine demonstrated protection against severe outcomes of secondary DENV infection in pre-immune individuals, but not in DENV-naïve individuals (28), indicating that pre-existing immunity to a related flavivirus can influence vaccine efficacy. Interestingly, licensed vaccines against JEV, based on inactivated or live attenuated virus platforms, used in endemic regions of East, South and Southeast Asia, showed some level of immunity against DENV infection in a mouse model, as measured by neutralizing antibodies and T cells (29).

A large number of ZIKV vaccine candidates have been developed to date based on different vaccination platforms, including chimeric live-attenuated virus, plasmid DNA, purified-inactivated virus (ZPIV), adenovirus-vectored, and mRNA (30–34). Although some have advanced to phase 1 or 2 clinical trials, efficacy studies have been hampered by the declining incidence of infection, and so far no candidate has been licensed (35). A strategy for ZIKV vaccine distribution in regions where a high proportion of the population has been exposed to or vaccinated against other flaviviruses would need to consider the implications of pre-existing flavivirus immunity, as it pertains to the potential for priming or diminution of ZIKV-specific immune responses. The potential for a priming effect has been suggested from a study showing that vaccination with one or two doses of ZPIV vaccine induced higher titers of neutralizing antibodies in individuals pre-exposed to DENV compared with flavivirus-naïve individuals (36). Similarly, a clinical trial that tested a live-attenuated DENV-2 vaccine showed that YFV pre-immune individuals developed higher anti-DENV-2 neutralizing antibody titers than flavivirus-naïve individuals after DENV-2 vaccination (37). Little is known about the consequences of pre-existing flavivirus immunity on vaccine-induced T cell responses. Here, we explore cross-reactive T cells induced by a ZPIV vaccine in individuals pre-immunized with JEV or YFV vaccines to investigate the influence of pre-existing flavivirus immunity on the establishment of cellular immune response to a ZIKV vaccine.

## Materials and Methods

### Participants and PBMC Samples

PBMC samples used in this study were collected at the Walter Reed Army Institute of Research (WRAIR) from participants enrolled in a Phase 1, first-in-human, double-blinded, randomized, placebo-controlled trial of ZPIV with aluminum hydroxide adjuvant in healthy flavivirus-naive and flavivirus-primed adults (NCT02963909). Participants were screened for pre-existing antibodies against ZIKV, JEV, YFV, DENV, and WNV by microneutralization assay, and only seronegative individuals were enrolled. All participants included in this subsegment of the overall trial cohort received three doses of 5.0 μg ZPIV with aluminum hydroxide gel adjuvant at week 0, week 4, and week 28, as previously described (32). Only those subjects who received three doses of vaccine and who had adequate samples available for testing were included rather than the entire vaccinated cohort included in the clinical trial. Nine flavivirus-naïve participants were included in our study. Two to three months before the first ZPIV dose, twelve individuals in our study received a two-dose regimen of JEV vaccine (IXIARO®, Valneva) 28 days apart, and 12 individuals received one dose of YFV vaccine (YF-VAX®, Sanofi Pasteur). All peripheral blood samples were processed at the Specimen Processing Lab at WRAIR following standard operational procedure for PBMC isolation and stored frozen in liquid nitrogen until use.

The study protocol was approved by the local institutional review board. Written informed consent from all participants were obtained before screening. The clinical trial from which samples were obtained adhered to the policies for protection of human study participants, as prescribed in Army Regulation 70-25.

### Peptide Selection

The ZIKV polyprotein sequence from the Puerto Rico isolate (PRVABC59—GenBank AMC13911.1) was used to predict T cell epitopes from structural proteins (C, prM/M, and E). MHC class I epitopes were predicted by NetMHCpan version 3.0, and MHC class II epitopes were predicted by NetMHCII 2.2 Server, taking into account the most frequent HLA alleles in the population: HLA A^*^02, A^*^24, B^*^07, B^*^15, B^*^40 supertype alleles for MHC class I and HLA-DRB1^*^0101, ^*^0401, ^*^0701, ^*^1101, ^*^1302 for MHC class II. Peptides with 15 amino acids long overlapping by 11 amino acids were designed from ZIKV structural protein sequence, and those that contained predicted MHC class I and/or II epitopes with affinity <50 nM were selected.

The ZIKV polyprotein sequence was aligned to sequences from JEV strain SA14-14-2 (GenBank ARE67893.1) and from YFV strain 17D (GenBank AFQ32465.1) using Clustal Omega multiple sequence alignment to classify the selected peptides as flavivirus-conserved or non-conserved. Our classification considered 15-mers containing 8 or more (>50% of the peptide) identical amino acids or bearing strong property similarities among the 3 polyprotein sequences as flavivirus-conserved. Finally, we selected some variant peptides from JEV and YFV that comprised epitope sequences described previously (38–42). All the peptide sequences are listed on Supplementary Table 1.

### CD4 and CD8 T Cell Proliferation Assay

**Figure 1:**
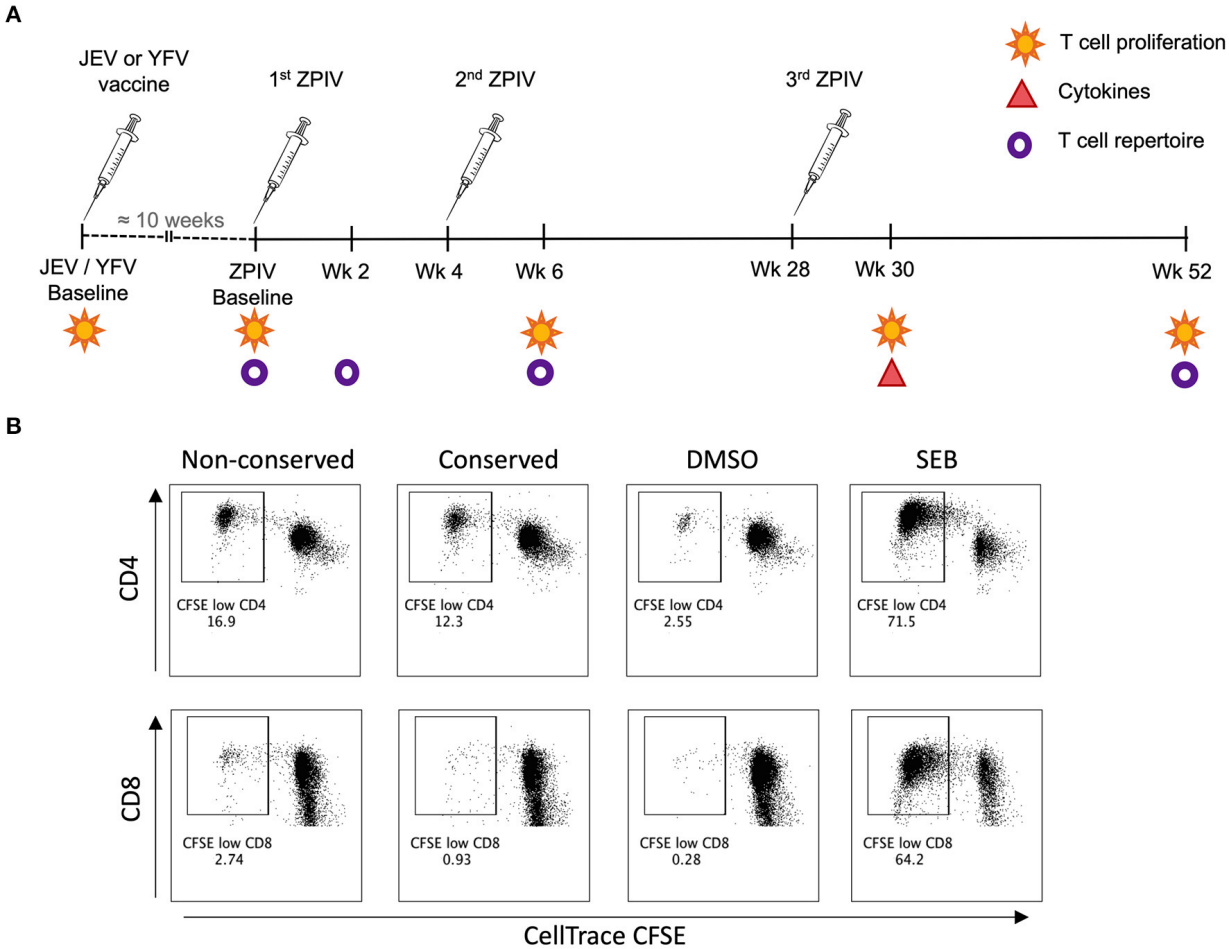
Methodology for T cell response analysis. **(A)** Vaccination timeline showing the analysis performed for each time-point; **(B)** Representative flow cytometry plots showing CD4 and CD8 T cell proliferation analysis based on CellTrace CFSE staining.

PBMCs were labeled with CellTrace™ CFSE (Invitrogen, USA) following manufacturer's instructions, washed extensively, and cultured in RPMI supplemented with 8% human serum (Access Biologicals, USA) for 6 days in the presence of peptide pools (1 μg/mL each) or whole viral particle from ZPIV. For the negative control, each sample was also cultured with DMSO using the same volume as the peptide pools. For the positive control, each sample was stimulated with 0.1 μg/mL SEB. After 3 days of culture, half of the media was changed. After 6 days, the supernatants were collected and frozen for posterior cytokine measurement, and the cells were stained with LIVE/DEAD™ Fixable Aqua (Invitrogen, USA) followed by surface markers (anti-CD3 APC, anti-CD8 BV785, anti-CD4 BV650, anti-CD56 BV510, anti-CD19 BV510, anti-CD14 BV510—Biolegend, USA). For analytical flow cytometry, cells were fixed with 2% paraformaldehyde (Sigma-Aldrich) at 4°C for at least 10 min and acquired on a flow cytometry machine (LSRII, BD Biosciences, USA). For TCR repertoire sequencing, unfixed cells were sorted on a BD FACSAria flow cytometry based on the markers LD^−^CD56^−^CD19^−^CD16^−^CD3^+^CD4^+^CFSE^low^. All flow cytometric results were analyzed using FlowJo software version 10.6.1 (Becton Dickinson & Company). T cell proliferation was calculated by subtracting the DMSO condition from stimulated cultures on LD^−^CD56^−^CD19^−^CD16^−^CD3^+^CD4^+^CFSE^low^ and LD^−^CD56^−^CD19^−^CD16^−^CD3^+^CD8^+^CFSE^low^ gates (shown in Figure 1B).

### Cytokine Measurement

A panel of cytokines, including IFN-γ, TNF, IL-4, IL-5, and IL-10, was measured using the Human Ultrasensitive Cytokine Magnetic 10-Plex Panel (Invitrogen, USA) from culture supernatants of PBMC samples from week 30 which were stimulated for 6 days with peptide pools or with DMSO negative control. The procedure was run according to manufacturer's instructions. A Bioplex-200 system was used to acquire samples, and the data was analyzed with the BioPlex Manager Software (Bio-Rad Laboratories, USA).

### TCR Repertoire Sequencing

Total RNA was extracted from sorted cells using RNAzol® RT (Molecular Research Center, USA), following manufacturer's instructions. TCR genes were sequenced using a method similar to one previously described (43). Briefly, cDNA was generated using an oligo (dT) primer and a template-switching oligo during an incubation at 42°C for 90 min then 70°C for 10 min. To amplify the V-CDR3-J region of the TCRβ chain, a PCR was performed with a pair of primers, one binding the SMARTer oligonucleotide region (5′-AAGCAGTGGTATCAACGCAGAGT-3′), and the other binding the TCRβ constant region (5′-TGCTTCTGATGGCTCAAACACAGCGACCT-3′) using the Kapa HiFi system (Kapa Biosystems, Wilmington, MA, USA) as follows: 95°C for 5 min; 5 cycles at 98°C for 15 s and 72°C for 1 min; 5 cycles at 98°C for 15 s, 70°C for 10 s and 72°C for 1 min; 20–30 cycles at 98°C for 15 s, 60°C for 10 s, and 72°C for 1 min. A second PCR was performed to add an Illumina adaptor and a unique index barcode to each individual sample. The cDNA and each PCR product were purified using the HighPrep™ PCR Clean-up System (MagBio, USA). Library size and quality were measured using an Agilent Bioanalyzer High Sensitivity DNA Chip (Agilent, USA) to verify the presence of a unique band of ≅650 bp. The library concentration was calculated using Kapa Illumina Library Quantification Assay (Kapa Biosystems, USA). All TCRβ library samples were pooled in the same proportion according to their cell numbers for high-throughput sequencing by paired end MiSeq (Illumina, USA) 2×151 base pair reads.

### TCR Repertoire Computational Analysis

Raw, paired-end Illumina reads were demultiplexed using an in-house Java program. MiXCR was used to assign TCRβ annotations for each read-pair and to count TCRβ clonotypes (44). Using a table of all clonotypes contained within all samples, pairwise Morisita-Horn similarity indices were calculated with the R package vegan (45), specifically comparing time-points and stimulations. The table of similarity distances was then hierarchically clustered using the UPGMA algorithm and plotted with gplot heatmap.2 in R. Circos was used to visualize reoccurring clonotypes in a circular format (46). The Shannon Diversity and species richness were calculated on a per-sample basis using the previously mentioned table of all clonotypes and samples with the R package vegan. Both Shannon diversity and species richness were normalized by calculating the maximum possible value, based on the input number of cells for each library, and dividing the observed value by the maximum value to give a value between 0 and 1. They were subsequently visualized in R with the packages ggplot2, ggpubr, and ggsignif. TCRβ chain CDR3 sequences were aligned with MUSCLE, alignment distances were calculated in R with SeqinR (47), and MDS were plotted with vegan, ggplot2, and ggfortify.

### Neutralizing Antibody Titers

Neutralizing antibodies were measured by a wild-type live-virus microneutralization assay as described previously (32).

### Statistical Analysis

All statistical analyses were performed in Prism 8 (GraphPad Software) or R (http://www.r-project.org/). Further details on statistical analysis are listed on the figure legends.

## Results

### Pre-vaccination With JEV Vaccine, but Not YFV Vaccine, Alters Duration of CD4 T Cell Memory to ZIKV

To determine the effect of pre-existing immunity to heterologous flaviviruses on the establishment of T cell responses to ZIKV, we studied samples from flavivirus-naïve and JEV or YFV pre-vaccinated individuals that received 3 doses of the ZPIV vaccine (sample details in section Materials and Methods). In order to assess T cell responses, we performed an *in silico* epitope prediction for CD4 and CD8 T cell epitopes and selected 97 peptides from ZIKV structural proteins that contained these predicted epitopes. These peptides were used to stimulate PBMC samples in two separate pools: flavivirus-conserved or non-conserved epitopes, according to their amino acid sequence similarity between the 3 virus vaccines included in this study: ZIKV, JEV, and YFV. For pre-vaccinated groups, PBMCs were also stimulated with a peptide pool from each correspondent pre-vaccination (JEV or YFV).

Traditionally, T cell responses are measured based on their cytokine production upon peptides or whole antigen stimulation by intracellular cytokine staining. However, this method is often not sensitive enough to detect responses to vaccines, since antigen-specific responses are masked by bystander cytokine production of memory T cells (48). Additionally, in order to stain intracellularly, the cells need to be fixed with paraformaldehyde, which impairs downstream RNA sequencing methods. To overcome these limitations, we measured antigen-specific T cell responses based on their proliferation after peptide stimulation. This strategy allowed us to sort antigen-specific T cells for RNA sequencing to explore their T cell receptor (TCR) repertoire.

**Figure 2:**
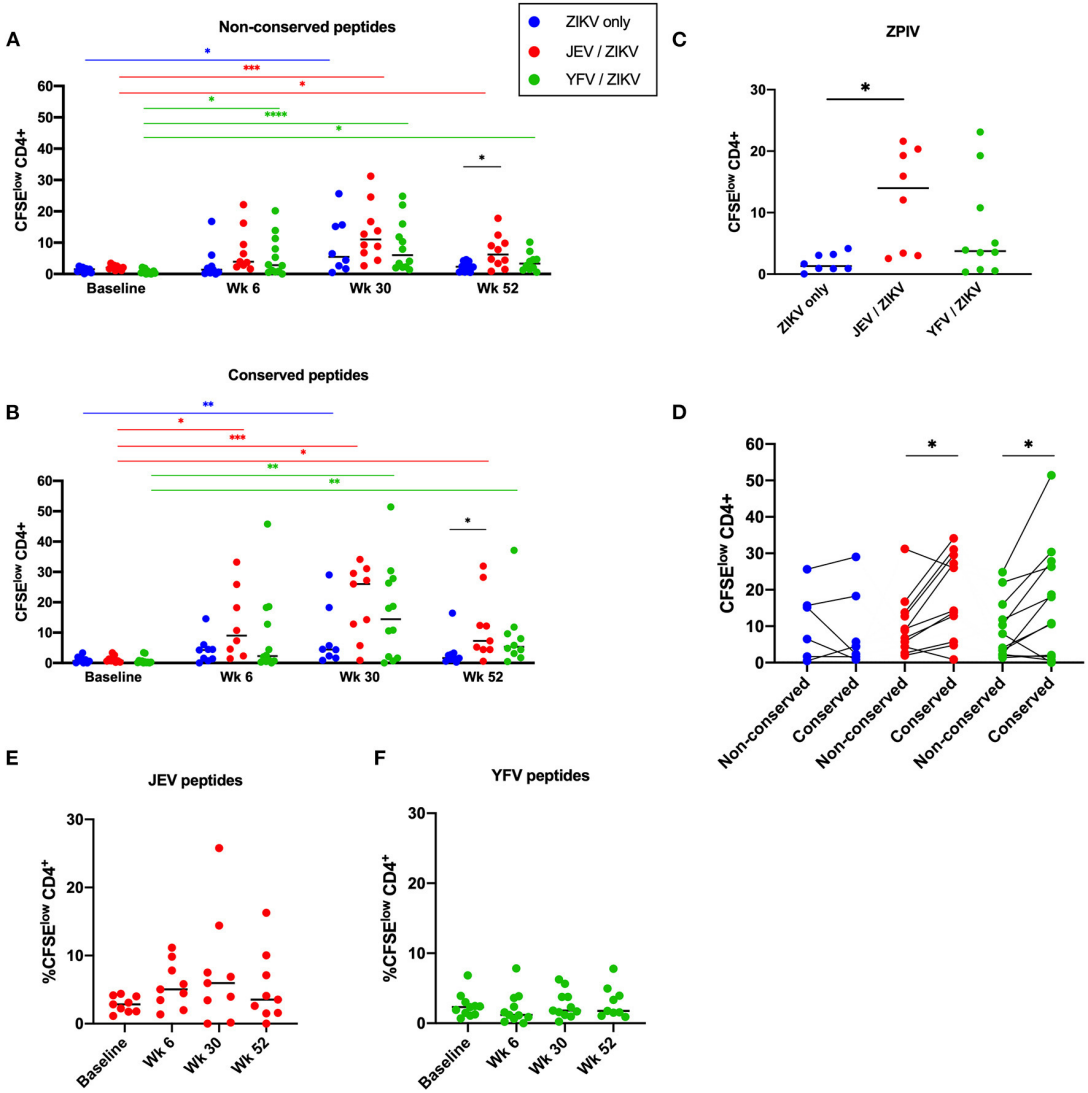
Pre-vaccination with JEV vaccine, but not YFV vaccine, alters duration of CD4 T cell memory to ZIKV. CD4 T cell responses against non-conserved **(A)** and flavivirus conserved **(B)** peptides from ZIKV structural proteins. **(C)** CD4 T cell responses against whole viral particle (ZPIV) at week 52. **(D)** Comparison of magnitudes of CD4 T cell responses at the peak time-point (week 30) between stimulations with different ZIKV peptide pools. **(E)** CD4 T cell responses against peptides from JEV structural proteins. **(F)** CD4 T cell responses against peptides from YFV structural and non-structural proteins. Statistical significance was determined by Kruskal-Wallis followed by Dunn's multiple comparison test, except for **(D)**, which Wilcoxon matched-pairs signed rank test was used. *P* values are represented as *P < 0.05, **P < 0.005, ***P < 0.0005, and ***P < 0.00005.

Using a CFSE-based assay, we assessed CD4 and CD8 T cell proliferation as a measure of T cell memory from PBMC samples collected at baseline (before pre-vaccination for JEV and YFV groups or before the 1st ZPIV for flavivirus-naïve group), week 6 (2 weeks after 2nd ZPIV dose), week 30 (2 weeks after 3rd ZPIV dose), and week 52 (latest time-point collected, 24 weeks after 3rd ZPIV dose) (Figure 1A). The frequency of CFSE^low^ population was used to measure magnitudes of responses (Figure 1B). Overall, we detected robust CD4 T cell responses starting at week 6 for both non-conserved and conserved ZIKV peptide pools (Figures 2A,B), but very low magnitudes of CD8 T cell responses toward both ZIKV peptide pools and only among a few individuals (Supplementary Figures 1A,B). The flavivirus-naïve group showed a significant increase in CD4 T cell responses at week 30 compared with baseline (*P* = 0.0291 for non-conserved epitopes; *P* = 0.0069 for conserved epitopes) with no significant difference in magnitude when compared with the other groups at this time-point. Earlier increases in CD4 T cell responses were detected at week 6 (after 2nd ZPIV dose) toward non-conserved epitopes for the YFV pre-vaccinated group (*P* = 0.019) and toward conserved epitopes for JEV pre-vaccinated group (*P* = 0.0113). Furthermore, JEV pre-vaccinated group showed more durable CD4 T cell responses with magnitudes significantly higher than the flavivirus-naïve group for both ZIKV peptide pools at week 52 (*P* = 0.0486 for non-conserved epitopes; *P* = 0.0323 for conserved epitopes) (Figures 2A,B). To confirm these results, we stimulated PBMC samples from week 52 with whole viral particles from the ZPIV vaccine and detected minimal CD4 T cell proliferation from the individuals that received only ZPIV, while JEV pre-vaccinated individuals showed significantly higher CD4 T cell responses (Figure 2C; *P* = 0.0324). Altogether these results suggest that pre-existing flavivirus immunity induced by a JEV inactivated vaccine may prime cross-reactive CD4 T cell responses to ZIKV, resulting in more durable responses to ZPIV vaccine.

We further compared the magnitude of ZIKV-specific CD4 and CD8 T cell responses toward conserved and non-conserved epitopes at the peak time-point (week 30) to determine if flavivirus pre-immunity would have an impact on immunodominance. While the flavivirus-naïve group showed no predominance in CD4 T cell responses toward either non-conserved or conserved epitopes, both pre-vaccinated groups showed significantly higher CD4 T cell responses toward flavivirus conserved epitopes (Figure 2D; *p* = 0.0244 for JEV pre-vaccinated group; *p* = 0.0342 for YFV pre-vaccinated group), showing that these epitopes are favored by cross-reactive responses induced by heterologous vaccinations.

We also measured CD4 T cell responses against JEV or YFV peptides from each correspondent pre-vaccination groups. Starting from week 6 (which is 4 months after the pre-vaccination) we detected low magnitudes of response for all time-points measured, with no significant increase after ZPIV vaccination (Figures 2E,F). Regarding CD8 T cells, the JEV pre-vaccinated group exhibited a very low magnitude of response to JEV epitopes (Supplementary Figure 1C), as expected for an inactivated-vaccine, while the live-atenuated YFV pre-vaccination induced robust and durable CD8 T cell responses toward YFV epitopes in some individuals (Supplementary Figure 1D). Collectively these data demonstrate that ZPIV vaccination did not boost CD4 T cell responses against the heterologous vaccines and, although YFV vaccine elicited robust YFV-specific CD8 T cell responses, it did not influence CD8 T cell responses against ZPIV.

### Th1 and Th2 Cytokines Are Produced in Individuals Pre-vaccinated With JEV Vaccine

**Figure 3:**
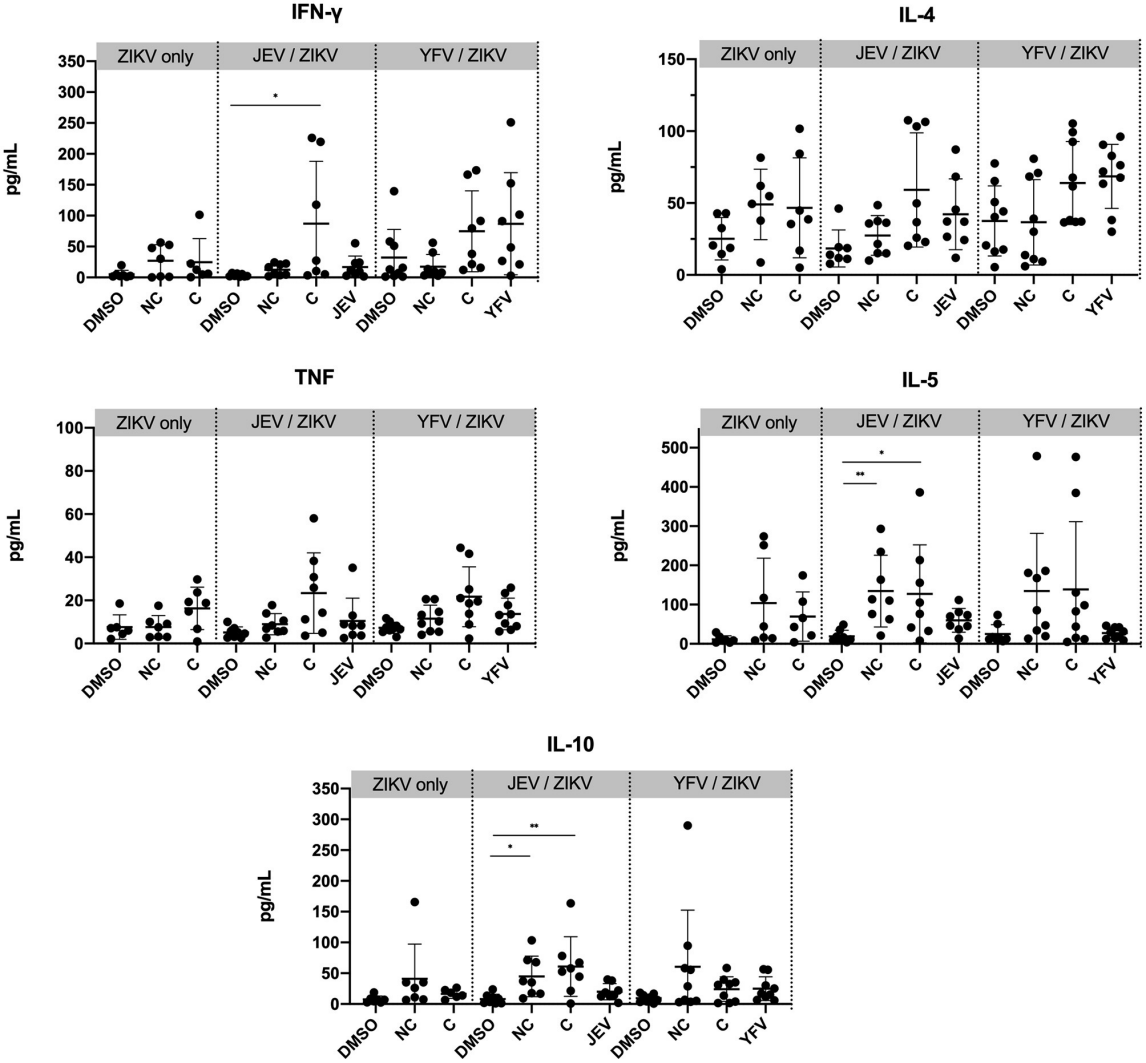
Greater cytokine production in PBMC from individuals pre-vaccinated with JEV vaccine. Cytokine concentrations were measured from the supernatant of PBMC samples from week 30 after 6 days of stimulation with peptide pools or DMSO (negative control). Statistical significance was determined by Kruskal-Wallis followed by Dunn's multiple comparison test. NC, non-conserved; C, conserved peptides. *P* values are represented as *P < 0.05 and **P < 0.005.

We next sought to investigate whether different vaccination groups would establish different types of T cell responses. We measured concentrations of the cytokines IFN-γ, TNF, IL-4, IL-5, and IL-10 in the supernatant of bulk PBMC cultures from peak time-point of CD4 T cell responses (week 30) after peptide stimulation for 6 days with either non-conserved or conserved ZIKV peptide pools, JEV or YFV peptides for each corresponding group, and DMSO as a negative control (Figure 3). We observed that PBMCs from individuals pre-vaccinated with JEV vaccine showed a significant increase in production of IFN-γ when stimulated with ZIKV conserved peptides and significant increases in IL-5 and IL-10 when stimulated with ZIKV conserved and non-conserved peptides as compared to non-stimulated controls (DMSO). No significant increase in cytokine release was detected in flavivirus-naïve or YFV pre-vaccinated individuals, and no significant differences were found when we compared each stimulation condition from the pre-vaccinated groups to the same stimulation condition in the ZIKV-only group. Although we cannot determine the cell source of cytokine release in the supernatant, these results indicate that the JEV pre-vaccinated group established a Th1- and Th2-like response to ZPIV.

### Cross-Reactive CD4 T Cell Clonotypes Elicited by Heterologous Vaccinations

Since our data indicated that pre-vaccination with JEV or YFV vaccines changed immunodominance of CD4 T cell responses to conserved epitopes, we wanted to investigate whether cross-reactive clonotypes are expanded by heterologous vaccinations. We selected 6 participants representative from each pre-vaccination group and analyzed samples from 3 months after pre-vaccination but before ZPIV vaccine (week 0, when all responses toward ZIKV epitopes are cross-reactive responses induced by the heterologous pre-vaccination), and the latest time-point after 3 ZPIV doses (week 52). Using the same CFSE-based assay described to measure T cell proliferation, we sorted CFSE^low^ CD4 T cells from PBMC stimulated for 6 days with ZIKV peptides (non-conserved and conserved pools) or correspondent pre-vaccination peptides (JEV or YFV) and sequenced their TCR beta chain.

**Figure 4:**
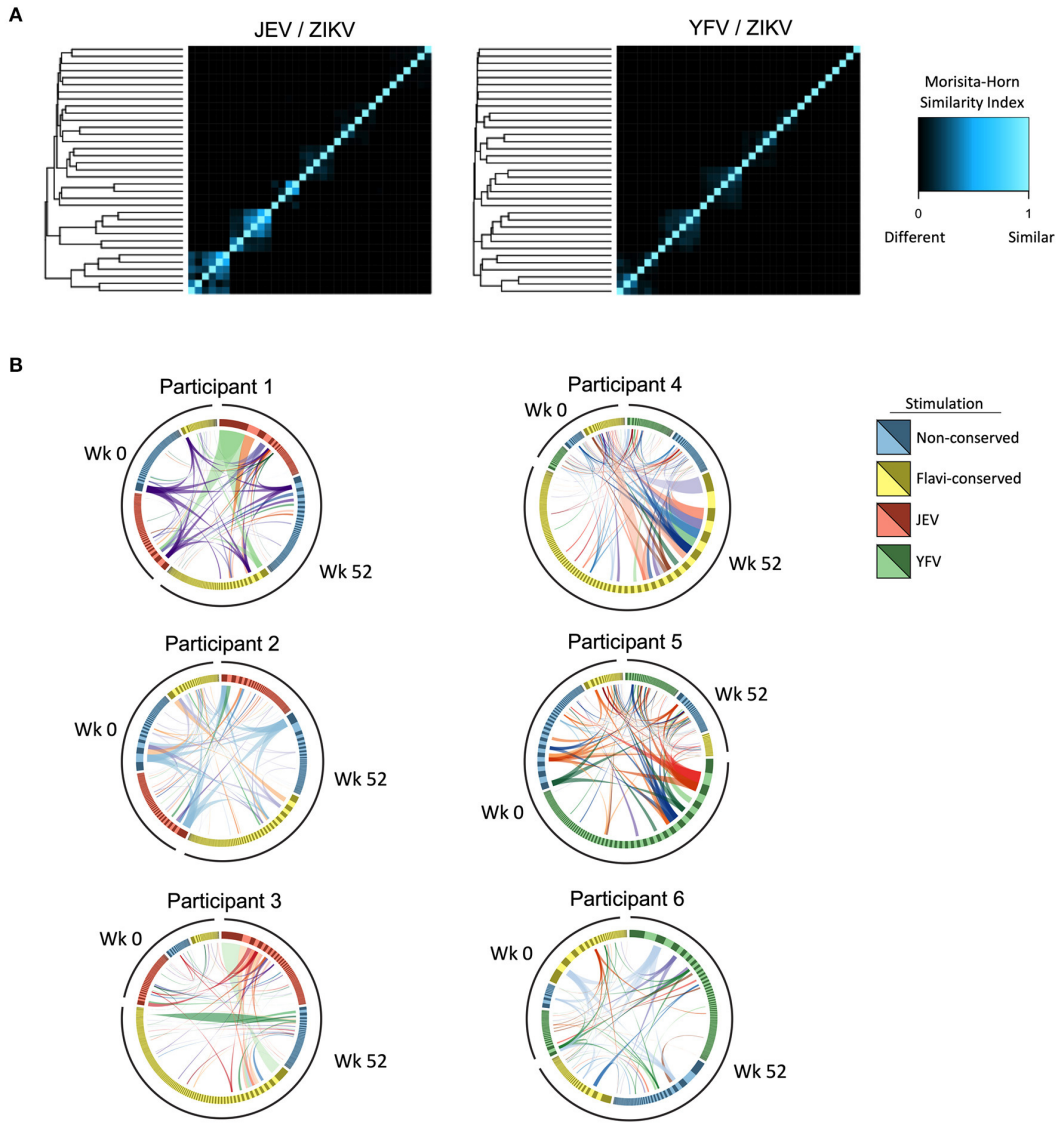
Cross-reactive CD4 T cell clonotypes elicited by heterologous vaccinations. **(A)** Morisita-Horn index was calculated to assess the similarity of TCR repertoire of CD4 T cells from before (week 0) and after 3 doses of ZPIV vaccination (week 52) responding to stimulation with peptides from ZIKV proteins (non-conserved and flavivirus-conserved peptides) and peptides from correspondent pre-vaccination (JEV—left panel or YFV—right panel). The heatmap shows the level of similarity between samples in each group compared against each other plotted as columns and rows, hierarchically clustered based on their similarity distances. **(B)** Circos plots show CD4 T cell clonotypes from participants with highest TCR repertoire similarity in groups pre-vaccinated with JEV (left circos plots) and YFV (right circos plots), in which the links represent identical clonotypes detected at different time-points and stimulations within each individual.

First, we calculated Morisita-Horn similarity indices to assess the similarity of TCR repertoires of samples from the different time-points and stimulations. Of note, in this analysis all samples from each participant clustered together and no similarity was found between different individuals, indicating that no public TCR was detected. In general, we observed higher TCR repertoire similarities within samples from different time-points and stimulations in individuals pre-vaccinated with JEV than those pre-vaccinated with YFV (Figure 4A), indicating the presence of more common clonotypes induced by both ZIKV and JEV peptide stimulation. For either groups, higher levels of TCR repertoire similarity did not associate with higher magnitudes of CD4 T cell responses (data not shown).

When we further analyzed the specificity of these overlapping clonotypes, we found that in the JEV pre-vaccinated group we detected the same clonotype in all samples analyzed from two participants with the highest TCR similarities (Figure 4B, participants 1 and 2, purple and blue links, respectively), indicating that cross-reactive clonotypes induced by JEV vaccination could react to both conserved and non-conserved ZIKV peptide pools and persist until week 52 after the 3 doses of ZPIV. Interestingly, TCR repertoire analysis from two participants from the JEV pre-vaccinated group showed that the most expanded clonotype responding to JEV peptides at week 52 was also the dominant clonotype responding to conserved epitopes from ZIKV (Figure 4B, participants 1 and 3, light green links), confirming the preferential expansion of cross-reactive clonotypes specific to ZIKV and JEV conserved epitopes. In the YFV pre-vaccinated group we found only few dominant overlapping clonotypes responding to both YFV and ZIKV epitopes, and those that were dominant in the response against YFV epitopes were usually subdominant in responses against ZIKV epitopes (Figure 4B, participants 4, 5, and 6). To further understand the similarity of CD4 clonotypes, we compared the CDR3 nucleotide sequences of the beta chains. Multidimensional scaling analysis of the aligned CDR3 regions showed that the most expanded clonotypes across different stimulations clustered and therefore were similar in sequence (Supplementary Figure 2). Considering that similar CDR3 sequences should bind to similar epitopes, this data corroborates the explanation that cross-reactive CD4 T cells were preferentially expanded. Together, these data suggest that both JEV and YFV pre-vaccinations induce cross-reactive CD4 T cells, but only those primed by JEV vaccine were preferentially expanded in response to ZIKV peptides after ZPIV vaccine.

### Diversity and Richness of TCR Repertoire Are Affected by Cross-Reactivity

**Figure 5:**
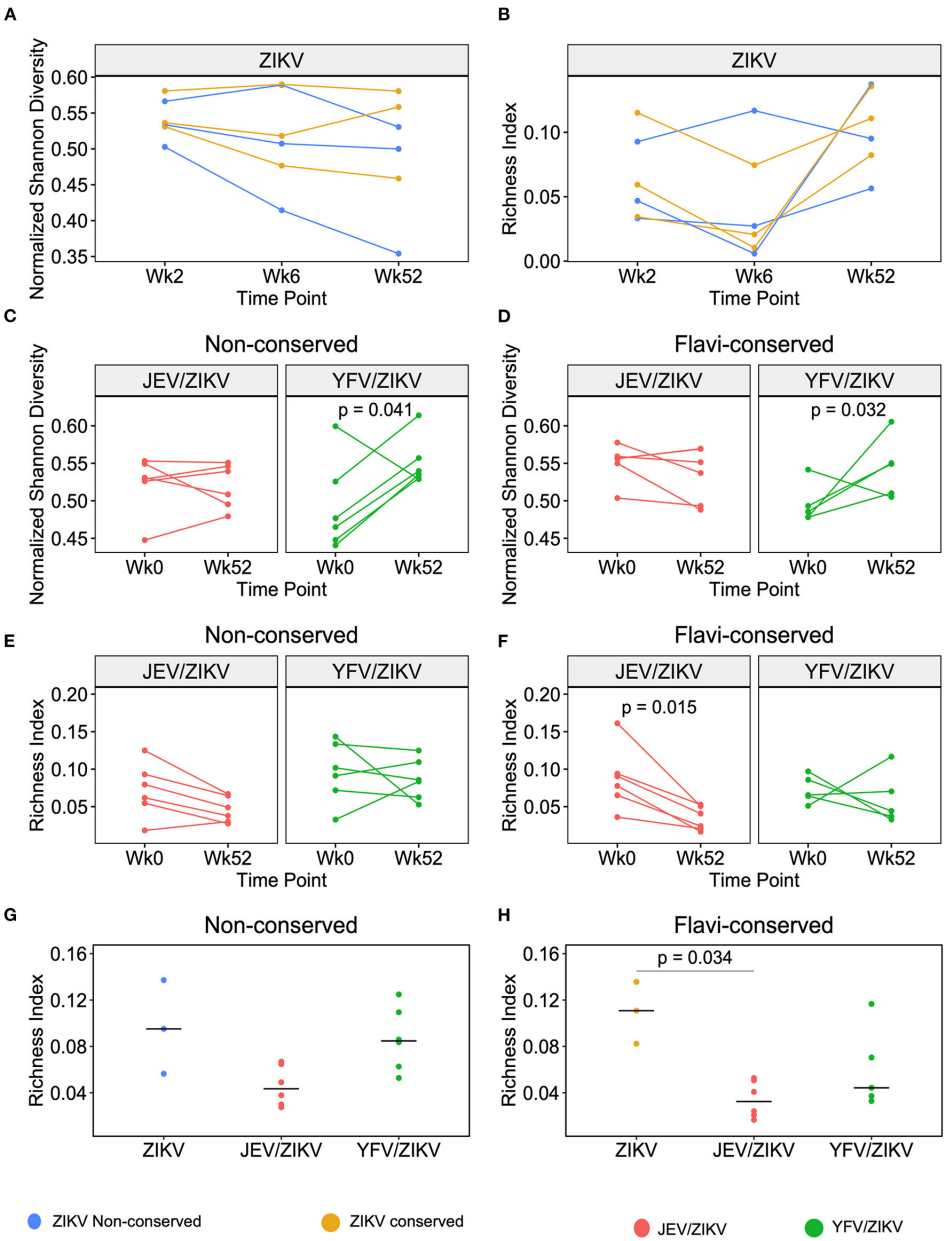
Richness and diversity changes in TCR repertoire of CFSE^low^ CD4 T cells after ZPIV vaccination. **(A)** Normalized Shannon Diversity index and **(B)** Richness index of TCR repertoire after stimulation with non-conserved (blue) or conserved (yellow) peptides from ZIKV proteins in flavivirus-naïve individuals vaccinated with ZPIV after 2 weeks post-1st dose (Week 2), 2 weeks post-2nd dose (Week 6) and 24 weeks post-3rd dose (week 52) of ZPIV vaccine. Normalized Shannon Diversity index of TCR repertoire after stimulation with non-conserved **(C)** or flavivirus-conserved **(D)** peptides from ZIKV proteins in individuals pre-vaccinated with JEV (red) or YFV (green). Richness index of TCR repertoire after stimulation with non-conserved **(E)** or flavivirus-conserved **(F)** peptides from ZIKV proteins in individuals pre-vaccinated with JEV (red) or YFV (green). **(G,H)** Richness index comparison between different vaccination groups at week 52. Statistical significance was determined by Wilcoxon matched-pairs signed rank test for **(C–F)**, or by Kruskal-Wallis followed by Dunn's multiple comparison test for **(G,H)**.

We next verified if cross-reactive responses induced by heterologous flavivirus pre-vaccination would influence the diversity and richness of CD4 T cell repertoire toward ZIKV epitopes after ZPIV vaccination. First, we analyzed the composition of the TCR repertoire of sorted CFSE^low^ CD4 T cells upon stimulation with non-conserved and conserved ZIKV peptides in 3 flavivirus-naïve individuals that received only ZPIV vaccine. In this group, we did not observe significant changes in TCR repertoire diversity or richness from the 1st (week 2) to 2nd ZPIV dose (week 6) and at the latest time-point after 3rd ZPIV dose (week 52) (Figures 5A,B), indicating that repeated doses of the ZPIV vaccine in flavivirus-naïve individuals do not impact the repertoire of CD4 T cell specific to ZIKV. In contrast, when we compared the TCR repertoire of CD4 T cells induced by pre-vaccination (week 0) and post 3 ZPIV doses (week 52), we observed a significant increase in the diversity of cells responding to both non-conserved and conserved epitopes in the YFV pre-vaccinated group, but not in the JEV pre-vaccinated group (Figures 5C,D). This result suggests that the ZPIV induces expansion of clonotypes different than the cross-reactive clonotypes induced by YFV pre-vaccination, increasing the TCR diversity after ZPIV administration. We hypothesize that in JEV pre-vaccinated individuals, the same cross-reactive clonotypes induced by pre-vaccination are expanded after ZPIV, resulting in no significant changes in TCR diversity similarly to the flavivirus-naïve group that received only ZPIV. To confirm the preferential expansion of cross-reactive clonotypes, we compared the richness of the TCR repertoire from before and after ZPIV vaccination, that reflects the total number of different clonotypes detected in the sample (Figures 5E,F). Interestingly, we observed a significant decrease in repertoire richness for CD4 T cells responding to conserved epitopes of ZIKV in the JEV pre-vaccinated group (Figure 5F), suggesting that a reduced number of clonotypes specific to conserved epitopes are expanded after ZPIV vaccination and persist until week 52. In fact, the richness index of TCR repertoire at week 52 was the lowest for the JEV pre-vaccinated group when compared with the same time-point in the other vaccination groups (Figures 5G,H), and significantly lower than the flavivirus-naïve group vaccinated only with ZPIV regarding CD4 T cells responding to conserved epitopes (Figure 5H). Altogether, these data indicate that cross-reactive CD4 T cell responses induced by JEV pre-vaccination had a greater effect than YFV pre-vaccination in shaping T cell responses to ZPIV vaccine.

### CD4 T Cell Responses From JEV Pre-vaccinated Group Correlate With Neutralizing Antibody Titers

**Figure 6:**
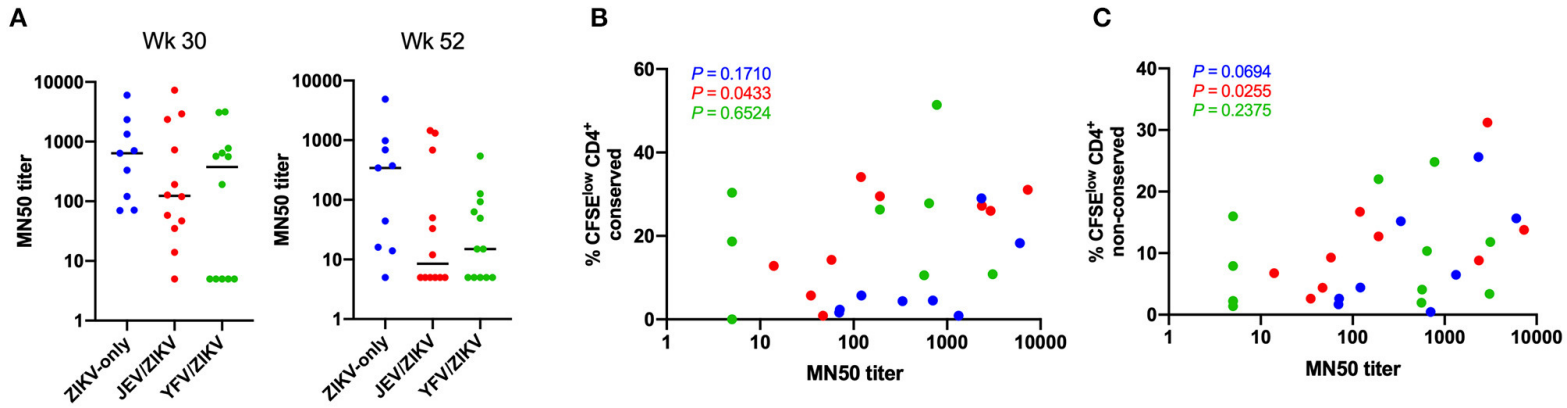
Correlation of ZIKV neutralizing antibody titers with CD4 T cell responses. **(A)** ZIKV microneutralization titers at week 30 (peak) and 52 (last time-point). **(B)** Correlation of microneutralization titers and CD4 T cell responses against non-conserved epitopes at week 30. **(C)** Correlation of microneutralization titers and CD4 T cell responses against conserved epitopes at week 30. In **(B,C)**, *P-*values for each group are shown with same color code from **(A)**.

Since CD4 T cell responses have been shown to play an important role in generating neutralizing antibodies against ZIKV (9, 10), we investigated whether the cross-reactive T cells observed in the pre-vaccinated groups would have any consequence in the production of neutralizing antibodies. There was no significant difference in ZIKV microneutralization titers between the vaccine groups at either week 30 (peak time-point) or week 52 (last time-point) (Figure 6A), but we observed a decreasing trend in these titers in both pre-vaccinated groups compared to flavivirus-naïve group at week 52. Interestingly, we found a significant correlation between these titers and CD4 T cell responses for conserved and non-conserved epitopes in JEV pre-vaccinated group at week 30, when antibodies and T cell responses peaked (Figures 6B,C). This correlation did not persist until week 52, when both types of responses waned. These data suggest that the generation of high titers of neutralizing antibodies in the JEV pre-vaccinated group may be more dependent on strong CD4 T cell responses. However, the flavivirus-naïve group was able to establish high titers of neutralizing antibodies in a manner slightly more independent of CD4 T cells, since we did not observe a significant correlation and the microneutralization titers remained high until week 52 in this group. Intriguingly, the YFV pre-vaccinated group included individuals that did not seroconvert and individuals that were able to produce neutralizing antibodies with titers similar to the other groups, but that phenomenon was not associated with the magnitudes of CD4 T cell responses.

## Discussion

The development of a protective vaccine against ZIKV infection is paramount to prevent future outbreaks. Due to the high antigenic similarity between ZIKV and other flaviviruses that co-circulate in the same area, newly developed ZIKV vaccines need to be studied for cross-reactive immune responses between related flaviviruses. This study demonstrates that the ZPIV vaccine, tested in a phase I clinical trial, elicits robust CD4 T cell responses to ZIKV structural proteins epitopes, and that prior immunization with JEV vaccine, but not with YFV vaccine, influences the duration of these responses. These differences were explained by the presence of CD4 T cell clonotypes induced by the JEV pre-vaccination that could cross-recognize ZIKV epitopes, resulting in preferential expansion of cross-reactive CD4 T cells and higher magnitudes of response at the latest time-point after 3 doses of ZPIV, indicating a longer durability. Although we detected the presence of cross-reactive clonotypes for ZIKV and YFV epitopes in individuals pre-vaccinated with YFV, these clonotypes were subdominant in response toward ZIKV epitopes. Additional boosts of ZPIV after YFV pre-vaccination induced expansion of new ZIKV-specific CD4 T cell clonotypes, suggesting that responses to YFV and ZIKV vaccines are more virus-specific than cross-reactive. Furthermore, we detected skewed CD4 T cell responses toward conserved epitopes in both JEV and YFV pre-vaccinated groups, demonstrating some level of cross-reactivity *in vitro*, although the recalled cross-reactive memory response *in vivo* was only dominant for the JEV pre-vaccinated group.

The fact that JEV has higher sequence homology than YFV to ZIKV (17) could have accounted for the stronger priming effect of this vaccine preceding the ZPIV vaccination. Another factor that may have contributed is the vaccine composition. The JEV vaccine used in this study is based on purified-inactivated virus, similar to the ZPIV vaccine, while the YFV vaccine is a live-attenuated virus. The JEV inactivated vaccine comprises antigens from structural proteins of the virus, and was described to establish CD4 T cell responses, mainly toward E protein epitopes. These epitopes were shown by others to cross-react with DENV and WNV (49), as well as with ZIKV in our study. Pre-clinical studies of the ZPIV vaccine in rhesus macaques detected T cell responses primarily to ZIKV E protein following ZPIV vaccination, showing a similar immunodominance as the JEV vaccine (50, 51). On the other hand, the live-attenuated YFV vaccine encodes structural and non-structural proteins, resulting in a distinct immunodominance pattern, as the most frequent CD4 T cell epitopes were found as NS1 > NS3 > capsid > envelope > NS5 (52). Thus, this vaccine may induce immunodominant responses toward non-structural proteins at the expense of structural antigens that have homology with those presented by the ZPIV vaccine. Our study also suggests that the presence of non-structural antigens or active virus replication may be needed to induce CD8 T cell responses, as robust CD8 T cell activation has been demonstrated upon ZIKV infection in human and mice studies (53, 54), and cross-reactive CD8 T cell responses between a live attenuated JEV vaccine and ZIKV infection were described (55), but this type of response was not elicited by the ZPIV vaccine. The live-attenuated YFV vaccine is known to induce robust CD8 T cell responses (56). This robust response did not appear to sufficiently prime cross-reactive CD8 T cells that could improve responses to ZPIV in our study. Consistent with this are observations that CD8 T cells induced by the YFV vaccine are specific to YFV epitopes with minimal cross-reactivity to other flaviviruses (24).

Traditional methods for identifying immunodominant and cross-reactive epitopes through deconvolution of peptide pools require a large number of cells. Due to sample size limitations in our study, we were not able to search which individual peptides were driving the cross-reactive T cell responses. Instead, we used a TCR sequencing approach to identify cross-reactive clonotypes to peptide pools from different flaviviruses. Moreover, alignment of the CDR3 regions within the TCR repertoire of each individual could suggest similarities between epitope-specificity of dominant or subdominant clonotypes. Our method also allowed us to detect preferential expansion of CD4 T cell clonotypes primed by JEV pre-vaccination. This could be caused by “original antigenic sin,” in which memory T cells induced by the prior immunization respond more rapidly than naïve cells and, therefore, dominate the response to similar epitopes. This phenomenon has been demonstrated to induce T cells with lower avidity to variant peptides and produce an altered cytokines profile with weak effector functions, which has been associated with increased disease severity in DENV infections (57). A study of cross-reactive CD4 T cell responses between ZIKV and other flaviviruses in HLA-class II transgenic mice showed that CD4 T cells from ZIKV-immunized mice responded to related DENV4 epitopes with higher IL-17A production, while IL-10 production did not differ between ZIKV-specific and cross-reactive responses (21). IL-10 is an immunoregulatory cytokine produced during the acute phase of immune responses, and is involved in the prevention of tissue damage by inhibiting pro-inflammatory responses (58). IL-10 production by CD4 T cells was also detected in ZIKV-infected immunocompromised mice and was not associated with regulatory T cell phenotype, but rather with Th1 effector cells (9). In our study we detected release of IL-10, IL-5, and IFN-γ from PBMC of ZPIV vaccinated individuals, especially in individuals pre-vaccinated with JEV, indicating that the ZPIV-vaccination elicited Th1 and Th2 responses. In addition, we did not see evidence of a detrimental effect of the CD4 T cell cross-reactivity in the induction of neutralizing antibodies.

Higher antibody neutralization potency for ZIKV and higher cross-neutralizing antibody titers were found in DENV pre-immune individuals (22, 36, 53), suggesting a better quality of antibody response to ZIKV in DENV-immune than in flavivirus-naïve individuals. In contrast, our study showed that pre-existing immunity to JEV and YFV did not improve the titers of neutralizing antibodies against ZIKV. Antibody cross-reactivity between flaviviruses has also been associated with antibody dependent enhancement of infection, in which antibodies induced by one flavivirus can cross-react but poorly neutralize a similar flavivirus (59). This may explain the absence of ZIKV-neutralizing antibodies in several individuals pre-vaccinated with YFV in our study, though this lack of seroconversion did not preclude the establishment of ZIKV-specific CD4 T cell responses. Although not significant, there was a trend toward lower ZIKV-neutralization titers in JEV and YFV pre-vaccination groups, especially at week 52. These results were obtained with a subset of donors in the cohort, and the full data set including all donors is being analyzed for further investigations. The association of neutralizing antibody titers with CD4 T cell responses that we observed in JEV pre-vaccinated individuals may suggest that, if there was any impact of antibody cross-reactivity in the neutralization potency in this group, the CD4 T cell responses may have contributed to restore the quality of the humoral response, possibly by inducing more somatic hypermutations on cross-reactive B cells to improve neutralization potency of ZIKV-specific antibodies. In the absence of antibody cross-reactivity, such as in flavivirus-naïve individuals, CD4 T cell responses may contribute less to the generation of neutralizing antibodies.

Pre-exposure to other flaviviruses can affect the magnitude and epitope specificity of T cell responses to ZIKV upon natural infection or vaccination (20, 24). Our study showed that JEV pre-vaccinated individuals establish a more durable CD4 T cell response than do flavivirus-naïve individuals after ZPIV vaccination. However, the effect of pre-existing flavivirus immunity may have different consequences for antibodies and T cells, since we did not see a better persistence of neutralizing antibodies in the JEV pre-vaccinated group. Furthermore, the impacts of antibody and T cell cross-reactivities may be expected to be different regarding the antigen source such as from natural infection or vaccination with inactivated virus vaccines or live-attenuated vaccines and would warrant further investigation.

## Data Availability Statement

The original contributions presented in the study are publicly available. This data can be found here: http://www.ncbi.nlm.nih.gov/bioproject/701414. BioProject ID PRJNA701414.

## Ethics Statement

The studies involving human participants were reviewed and approved by Walter Reed Army Institute of Research Institutional Review Board. The patients/participants provided their written informed consent to participate in this study.

## Author Contributions

NL performed experiments, analyzed data, and wrote the manuscript. DM performed experiments and analyzed data. SD provided bioinformatics expertise. LL and MK were the principal investigators of the clinical trial. ST, KE, RJ, and RD are co-inventors of ZPIV. ST, NM, RJ, and KM developed the vaccine and designed the clinical trial. KM, DD, and NM provided expertise and feedback, and secured funding. LT provided expertise and feedback. All authors contributed to the article and approved the submitted version.

## Conflict of Interest

The authors declare that the research was conducted in the absence of any commercial or financial relationships that could be construed as a potential conflict of interest. The reviewer JB declared a past co-authorship with the following authors NM and KM, to the handling editor at the time of review.

## Supplementary Material

Supplementary Figure 1 Low magnitudes of CD8 T cell responses to ZPIV. CD8 T cell responses against non-conserved **(A)** and flavivirus conserved **(B)** peptides from ZIKV structural proteins. **(C)** CD8 T cell responses against peptides from JEV structural proteins. **(D)** CD8 T cell responses against peptides from YFV structural and non-structural proteins. Comparisons between groups and time-points did not reach statistical significance as determined by Kruskal-Wallis followed by Dunn's multiple comparison test.

Supplementary Figure 2 MDS of TCR beta CDR3 nucleotide sequences elicited by different stimulations. Color coding shown as in Figure 4B, in which blue = non-conserved; yellow = flavivirus conserved; red = JEV; and green = YFV epitopes.

Supplementary Table 1 Peptides list.
