# Supplementary figures and images for: Pre-existing Immunity to Japanese Encephalitis Virus Alters CD4 T Cell Responses to Zika Virus Inactivated Vaccine
Source: Front Immunol. 2021 Feb 24;12:640190. doi: 10.3389/fimmu.2021.640190 (PMC7943459; doi:10.3389/fimmu.2021.640190)

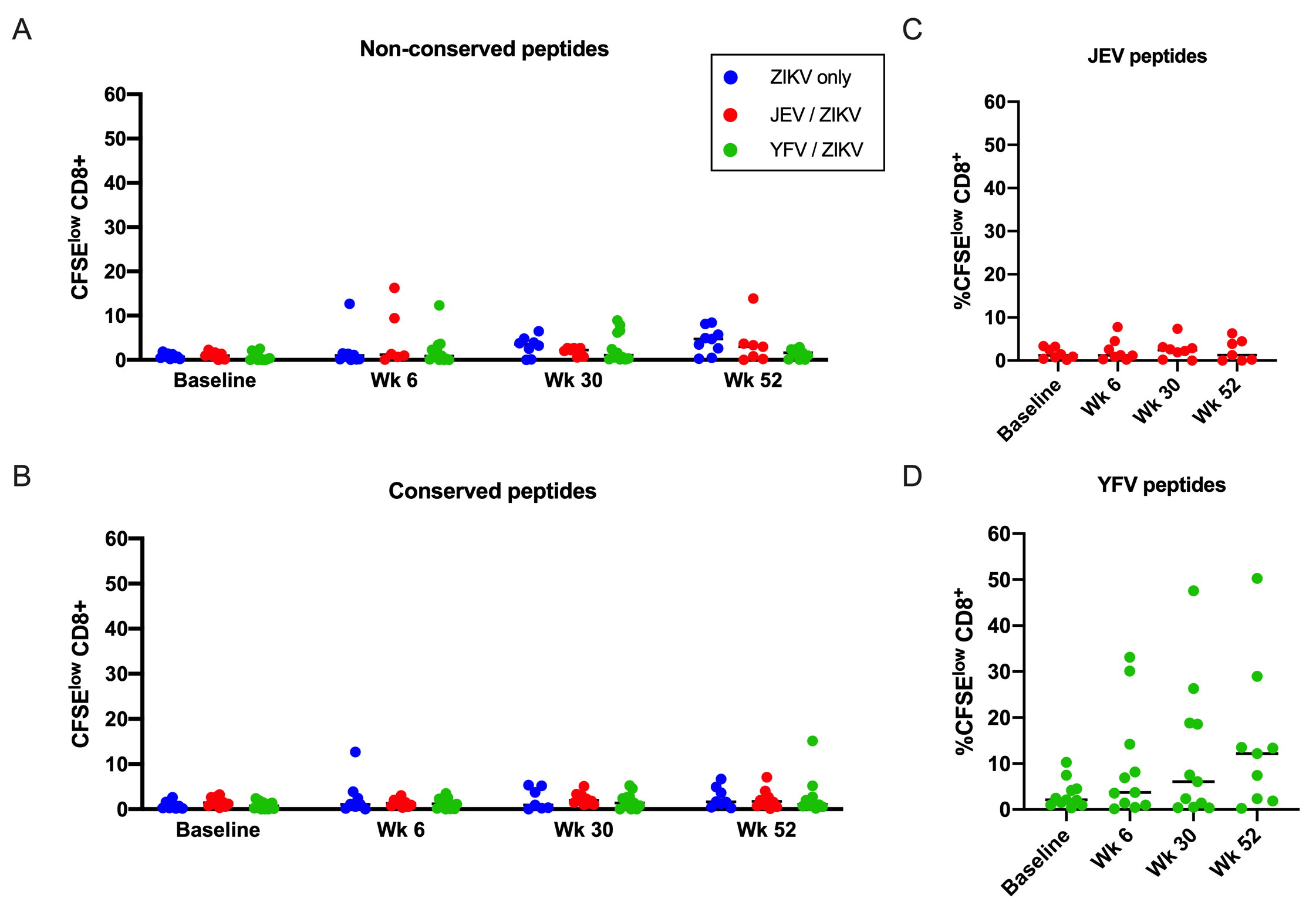

Supplement: Supplementary Figure 1 — Low magnitudes of CD8 T cell responses to ZPIV. CD8 T cell responses against non-conserved (A) and flavivirus conserved (B) peptides from ZIKV structural proteins. (C) CD8 T cell responses against peptides from JEV structural proteins. (D) CD8 T cell responses against peptides from YFV structural and non-structural proteins. Comparisons between groups and time-points did not reach statistical significance as determined by Kruskal-Wallis followed by Dunn's multiple comparison test. [file Image_1.TIFF]

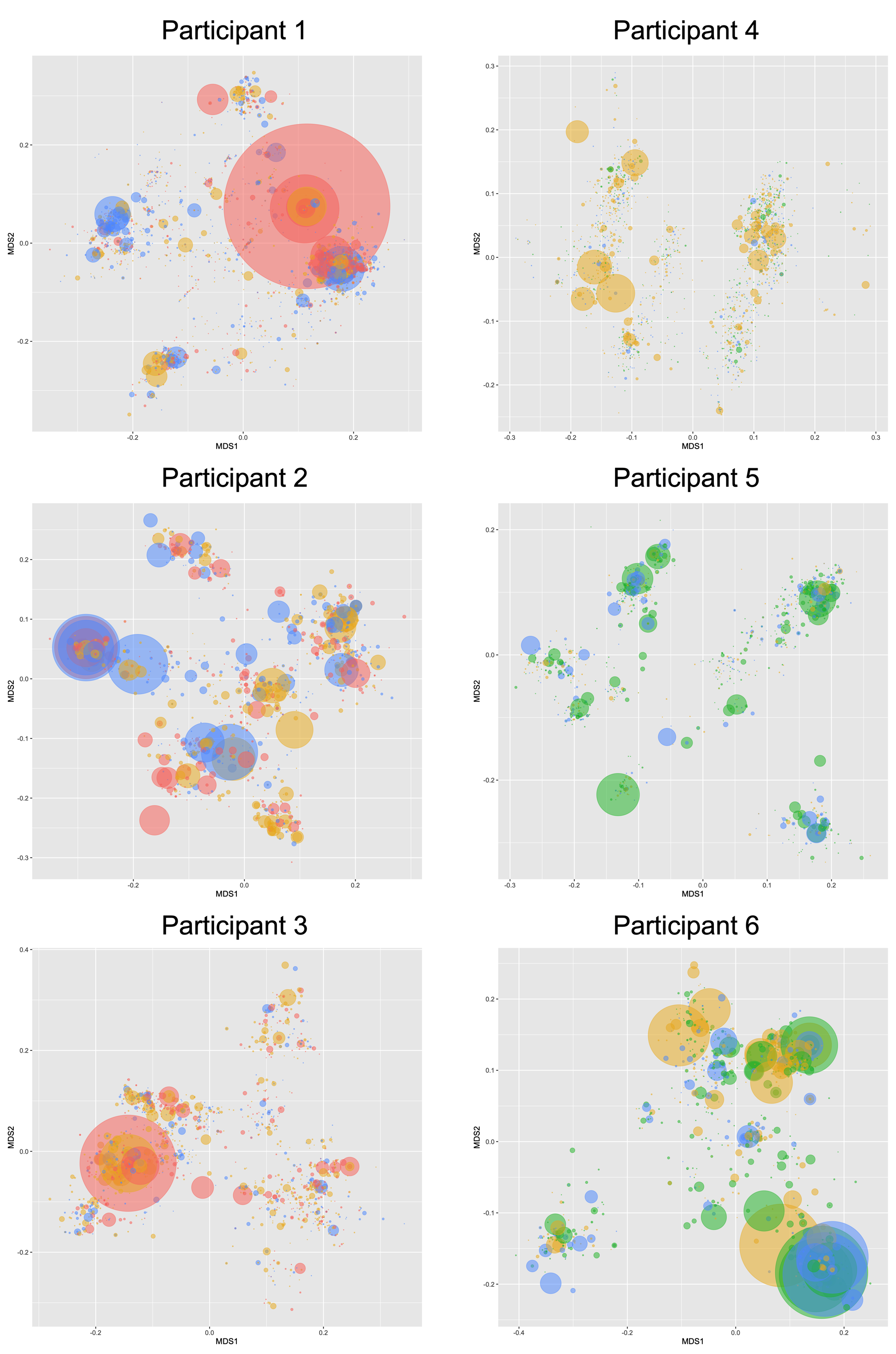

Supplement: Supplementary Figure 2 — MDS of TCR beta CDR3 nucleotide sequences elicited by different stimulations. Color coding shown as in Figure 4B, in which blue = non-conserved; yellow = flavivirus conserved; red = JEV; and green = YFV epitopes. [file Image_2.TIFF]
